# Predicting Patient Wait Times by Using Highly Deidentified Data in Mental Health Care: Enhanced Machine Learning Approach

**DOI:** 10.2196/38428

**Published:** 2022-08-09

**Authors:** Amir Rastpour, Carolyn McGregor

**Affiliations:** 1 Faculty of Business and Information Technology Ontario Tech University Oshawa, ON Canada; 2 Faculty of Engineering and Information Technology University of Technology Sydney Sydney Australia

**Keywords:** mental health care, outpatient clinics, wait time prediction, machine learning, random forest, system’s knowledge

## Abstract

**Background:**

Wait times impact patient satisfaction, treatment effectiveness, and the efficiency of care that the patients receive. Wait time prediction in mental health is a complex task and is affected by the difficulty in predicting the required number of treatment sessions for outpatients, high no-show rates, and the possibility of using group treatment sessions. The task of wait time analysis becomes even more challenging if the input data has low utility, which happens when the data is highly deidentified by removing both direct and quasi identifiers.

**Objective:**

The first aim of this study was to develop machine learning models to predict the wait time from referral to the first appointment for psychiatric outpatients by using real-time data. The second aim was to enhance the performance of these predictive models by utilizing the system’s knowledge while the input data were highly deidentified. The third aim was to identify the factors that drove long wait times, and the fourth aim was to build these models such that they were practical and easy-to-implement (and therefore, attractive to care providers).

**Methods:**

We analyzed retrospective highly deidentified administrative data from 8 outpatient clinics at Ontario Shores Centre for Mental Health Sciences in Canada by using 6 machine learning methods to predict the first appointment wait time for new outpatients. We used the system’s knowledge to mitigate the low utility of our data. The data included 4187 patients who received care through 30,342 appointments.

**Results:**

The average wait time varied widely between different types of mental health clinics. For more than half of the clinics, the average wait time was longer than 3 months. The number of scheduled appointments and the rate of no-shows varied widely among clinics. Despite these variations, the random forest method provided the minimum root mean square error values for 4 of the 8 clinics, and the second minimum root mean square error for the other 4 clinics. Utilizing the system’s knowledge increased the utility of our highly deidentified data and improved the predictive power of the models.

**Conclusions:**

The random forest method, enhanced with the system’s knowledge, provided reliable wait time predictions for new outpatients, regardless of low utility of the highly deidentified input data and the high variation in wait times across different clinics and patient types. The priority system was identified as a factor that contributed to long wait times, and a fast-track system was suggested as a potential solution.

## Introduction

The length and predictability of wait times are important factors that impact patient satisfaction, treatment effectiveness, and the efficiency of care that the patients receive. Providing patients with accurate wait time predictions and informing them about potential appointment delays increase the patients’ satisfaction level and enable care providers and staff members to manage the patient flow more effectively and efficiently [1-3]. Lengthy wait times are significantly associated with prognosis deterioration in mental health care [4] and are associated with higher rates of no-shows that adversely impact wait time management. The issue of long wait times is worse for children and youth with mental health problems, with some waiting as long as 2.5 years [5].

A great deal of research has been conducted on wait time prediction and identification of factors that drive lengthy wait times in physical health care sectors, including emergency departments [6], maternity emergency rooms [7], radiology departments [3], and oncology departments [8]. These wait time prediction models are usually developed for systems in which care is provided during a single visit to a care provider and the care is provided to patients individually. In contrast, mental health care is offered in a different context: the care is usually provided through multiple consecutive visits, the number of which is not necessarily known at the beginning of treatment, and the care can be provided to a group of patients, as in group consultation sessions. In addition, the psychiatric clinics also face a high rate of no-shows that make the task of wait time prediction even more difficult. Because of these intrinsic differences in the care provided for patients with physical problems and psychiatric patients, the wait time models developed for physical care cannot be readily used in the context of psychiatric care.

The task of predicting wait times becomes even more challenging when the available data are highly deidentified, that is, all direct identifiers (such as name, address, license plate) and quasi identifiers (such as gender, date of birth, zip code) are removed. Although it is a common practice to deidentify research data by removing the direct identifiers, the removal of quasi identifiers makes highly deidentified data more attractive from the privacy point of view, but at the same time, it compromises the utility of the information in the data that can otherwise be used to analyze and improve the system [9,10].

We use state-of-the-art machine learning (ML) methods to predict outpatient wait times at a tertiary mental health hospital by using real-time highly deidentified data. In addition, we use these models to identify key factors that drive the wait times. ML methods are sophisticated tools that can capture hidden patterns in large and imperfect data more effectively than conventional linear regression methods. ML methods are resistant to noise in the data and they quickly adapt to operational changes in wait time management processes without human supervision [11-14]. ML methods have been widely used in the mental health care sector for diagnosis [15-18], prognosis [19-22], treatment [23-25], and other medical purposes. A few literature review papers provide systematic surveys of these papers [26-29]. However, to the best of our knowledge, ML methods have not yet been applied to provide wait time prediction in the mental health care sector, although other health care sectors have benefitted from these sophisticated methods in their waiting list management.

A system’s knowledge is obtained through systems thinking, which is defined as seeing the relationship among components (rather than seeing the components individually) and observing the patterns of change (rather than static “snapshots”) [30]. In the context of an emergency department, it has been shown that ML models provide more accurate wait time predictions when they are enhanced with the system’s knowledge in the *presence* of quasi identifiers such as age and gender [31]. We obtain and use the system’s knowledge to enhance the predictive power of our ML models in the *absence* of quasi identifiers.

The first objective of this study was to develop 6 ML methods (namely, linear regression, random forest, weighted k-nearest neighbors, support vector machine, neural network, and decision tree) for real-time prediction of wait time for new outpatients in 8 outpatient clinics in Ontario Shores Centre for Mental Health Sciences (Ontario Shores) in Ontario, Canada. The second objective was to enhance the predictive power of ML models by using the system’s knowledge while having highly deidentified input data. The third objective was to assess variable importance to understand what factors drove long wait times. The fourth objective was to develop models such that care providers could understand and use them relatively easily without the need for background knowledge on ML models and their implementation.

## Methods

### Data Source and Data Preparation

In this research, we used highly deidentified retrospective administrative data from Ontario Shores to build ML models for predicting new outpatients’ wait times. Our focus was on 8 outpatient clinics, namely, Anxiety and Mood Disorders (AMD) Clinic, Traumatic Stress Clinic, Borderline Personality Self-Regulation Clinic, Women’s Clinic, Prompt Care Clinic, Prompt AMD, Prompt Transitional Aged Youth, and Prompt Adolescent Consultation. Our data included 4998 patients whose first appointment was between April 1, 2017 and September 30, 2019 (both days inclusive). We excluded 30 (0.6%) patients because of missing referral dates. Selection of patients based on the date of their first appointment, rather than their referral date, had caused a selection bias in favor of 2 groups of patients: (1) those who had longer wait times among patients whose referral date was just before April 1, 2017 and (2) those who had shorter wait times among patients whose referral date was just before September 30, 2019. To address this selection bias, we removed all patients whose referral date was before April 1, 2017, and for each clinic, we removed all patients whose referral date was after September 30, 2019 minus the 80th percentile of the wait times in that specific clinic. After removing the biased data, we were left with 4187 referral entries. Table 1 shows the breakdown of patient count by clinic.

**Table 1:** Summary statistics of patients’ wait time across different clinics (N=4187).

Clinic	Patients (n)	Mean (SD) days	Median days
Anxiety and Mood Disorders Clinic	298	98.08 (105.71)	64
Traumatic Stress Clinic	203	173.85 (113.58)	165
Borderline Personality Self-Regulation Clinic	181	107.42 (60.33)	112
Women’s Clinic	155	80.19 (47.08)	75
Prompt Care Clinic	2338	29.05 (23.90)	21
Prompt Anxiety and Mood Disorders Consultation	436	186.42 (138.16)	205.5
Prompt Transitional Aged Youth Consultation	402	54.5 (45.77)	37
Prompt Adolescent Consultation	174	97.14 (57.17)	90.5

### Variables

#### Outcome Variable

We aimed to predict the wait time, defined as the time from referral to the first appointment.

#### Predictor Variables Included in Our Data Set

Relevant variables for each patient were selected from the electronic health record. The medical variables included referral date, triage date, priority level (low, medium, and high) designated at triage, all appointment dates, status of each appointment (attended, no-show, and cancelled), and possible status changes while waiting or receiving care. 

#### Engineered Predictor Variables

**Figure 1:**
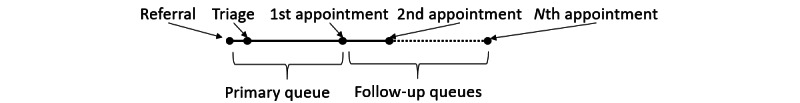
A schematic view of an outpatient receiving mental health care.

To better understand how our 8 clinics provided care to their patients, we had monthly feedback sessions with the care providers. In these sessions, components of the care system were identified and their interactions were outlined. Figure 1 presents a schematic view of the care processes at our clinics of interest. At first, care providers (usually family doctors) sent referrals to Ontario Shores. Then, a triage clinician assessed the patients and made intake decisions (accept or decline). Accepted patients entered the wait list associated with each clinic and remained there until the first appointment with their clinician. The outcome variable, wait time, was actually the wait time of the primary queue, as presented in Figure 1. Primary queue patients who were ahead of the new arrival directly impacted the new patient’s wait time. Understanding how the system worked led us to understand that although follow-up queues were downstream to the primary queue, they also indirectly impacted the wait time of the new patients. This indirect impact happened because the follow-up queues utilized the same resources (clinicians) that the primary queue utilized. Therefore, the new patient’s wait time depended on how much of the resource capacity was utilized by the follow-up queues. Another important output from our systems analysis discussions was that the clinics had utilizations close to 100%, which meant all of the care capacity offered by the clinics were assigned to patients. This helped us to approximate the offered care capacity by adding up the provided care. Obtaining the system’s knowledge led us to define and measure the following predictor variables: (1) number of patients from each priority level in primary and follow-up queues, (2) the accumulative wait time of patients from each priority level who were in follow-up queues, (3) the accumulative amount of service (treatment) that patients from each priority level in follow-up queues (note that the amount of service received in the primary queue is zero) had already received, (4) the accumulative amount of time patients from each priority level had already spent in follow-up queues, and (5) the total care capacity during 30-, 60-, and 90-day time windows just before the referral date.

### Missing Values

Some patients were missing their designated priority level at triage. However, those patients had a priority designated to them at a later date (possibly owing to re-evaluations while waiting). If a priority level at triage was missing, we replaced that with the priority level designated at the closest date after triage.

### Dimensionality Reduction

High dimensionality, that is, having too many variables in a model, may cause many complications, including overfitting and producing a higher sampling variance (ie, sensitivity to small fluctuations in the training set) [32-34]. We reduced the dimensionality of our data by selecting a subset of variables (and discarding the rest) while retaining as much information as possible from all variables. We kept all of the medical variables, and among variables obtained from systems analysis, we calculated the pairwise Pearson correlation and removed the redundant information if the correlation was larger than 90%, as in [35].

### Outliers

We used the generalized extreme studentized deviate method to identify the outliers [36]. This method iteratively applies the generalized extreme studentized deviate test and progressively evaluates anomalies by removing potential outliers and recalculating the test statistic and the associated critical value. The procedure continues until all outliers are identified.

### ML Methods

#### Implementation

We examined 6 different ML methods, namely, linear regression, random forest, weighted k-nearest neighbors, support vector machine, neural network, and decision tree [11]. We used R version 4.0.2 (2020-06-22) and developed all our predictive models in the tidymodels ecosystem of packages [37]. The tidymodels ecosystem was used to streamline the modeling procedure and to avoid coding variations caused by using separate packages for each ML tool. Streamlining the modeling procedure simplified the implementation and debugging steps and therefore made the models more likely to be used by Ontario Shores.

#### Tuning and Evaluation

For each of the ML modeling approaches, there were multiple hyperparameters that we needed to tune to make sure that the obtained output was the best (or close-to-best) possible from that model. To obtain good models and to avoid overfitting, data for each clinic were randomly divided into training (75% of the data) and testing (the remaining 25%) sets. First, we applied the Latin hypercube sampling method [38] to create the search grid within the range of values of each hyperparameter. Then, we selected the best value for each hyperparameter by conducting an exhaustive grid search using the 10-fold cross-validation method. As our outcome variable, wait time, was continuous, we used the root mean square error (RMSE) to compare the performance of different models.

### Ethics Approval

This study was approved by the Research Ethics Boards at Ontario Tech University (15596) and Ontario Shores (19-009-D).

## Results

### Summary Statistics

The summary statistics of our data for different clinics are shown in Table 1. The average wait time widely varied between the clinics from 29 days for Prompt Care Clinic to 186 days for Prompt AMD Consultation. For more than half of the clinics, the average wait time was longer than 3 months. We also calculated the median wait times, which varied between 21 days for Prompt Care Clinic and 205.5 days for Prompt AMD consultation, and confirmed long wait times. Our data also showed large standard deviation values, which varied between 23.9 days for Prompt Care Clinic and 138.16 days for Prompt AMD consultation. Having a high variation among patients’ wait times made it difficult for both care providers and patients to be able to plan ahead.

**Figure 2:**
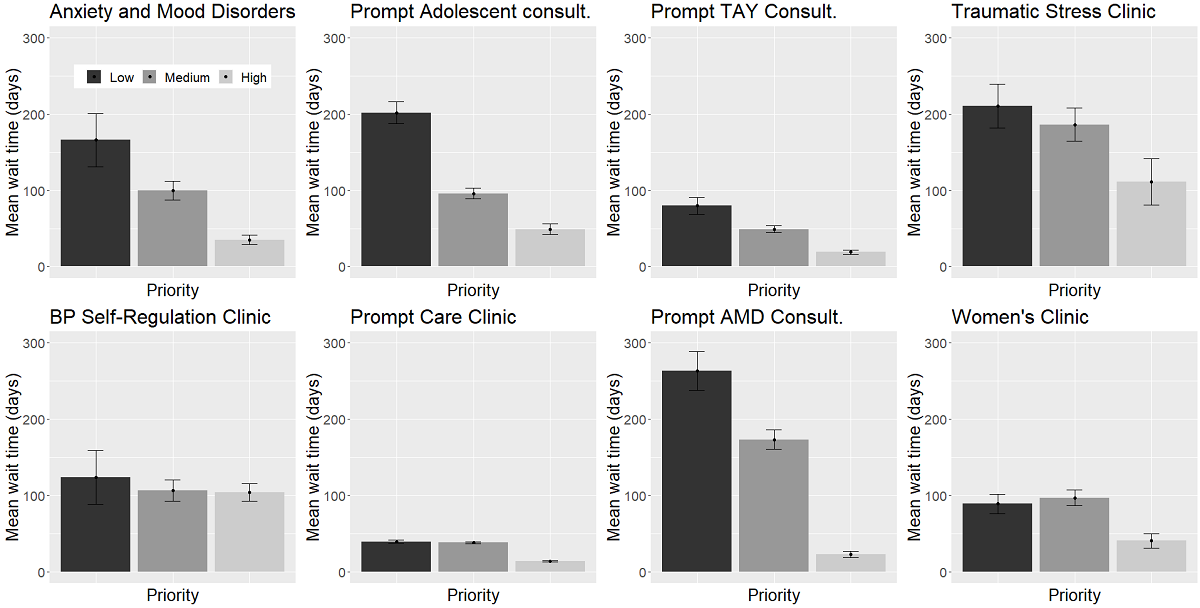
The mean wait time with 95% confidence interval by priority level and clinic. AMD: Anxiety and Mood Disorders; BP: Borderline Personality; Consult.: Consultation; TAY: Transitional Aged Youth.

The summary statistics of the appointments across different clinics are shown in Table 2. In total, the data set included 30,342 appointments out of which 4862 (16%) were no-shows. The number of scheduled appointments widely varied among clinics, from 307 appointments for Prompt Adolescent Consultation to 10,506 appointments for Borderline Personality Self-Regulation Clinic. The proportion of no-shows also widely varied across different clinics, from 2.9% (17/584) for Prompt AMD and Transitional Aged Youth Clinics to 22.2% (623/2804) for Women’s Clinic. Figure 2 shows the average wait time and 95% CI of all patients stratified by the priority level and clinic. This figure illustrates that low-priority patients had the longest wait time in all clinics, except for the Women’s Clinic, where the medium-priority patients had the longest average wait time.

**Table 2:** Summary statistics of all the appointments and no-show appointments per patient across clinics.

Clinic	All appointments (N=30,342)			No-show appointments (n=4862)		
	Patients (n)	Mean (SD)	Median	Patients, n (%)	Mean (SD)	Median
Anxiety and Mood Disorders Clinic	6830	21.68 (23.03)	17	1431 (20.9)	4.54 (6.84)	2
Traumatic Stress Clinic	5617	27.27 (16.08)	26	1148 (20.4)	5.57 (5.30)	4
Borderline Personality Self-Regulation Clinic	10,506	57.73 (50.18)	42.5	1453 (13.8)	7.98 (7.71)	5
Women’s Clinic	2804	17.97 (14.53)	17.5	623 (22.2)	3.99 (5.16)	2
Prompt Care Clinic	3167	1.34 (0.8)	1	158 (4.9)	0.07 (0.32)	0
Prompt Anxiety and Mood Disorders Consultation	584	1.08 (0.28)	1	17 (2.9)	0.03 (0.19)	0
Prompt Transitional Aged Youth Consultation	527	1.05 (0.22)	1	14 (2.6)	0.03 (0.18)	0
Prompt Adolescent Consultation	307	1.74 (2.3)	1	18 (5.8)	0.10 (0.37)	0

### Model Performance

We first applied our dimensionality reduction approach to the training set. Variables introduced in the section “Engineered Predictor Variables” appeared to contain similar information. We dropped all of them except for “Number of patients from each priority level in primary and follow-up queues.” We used these variables along with variables introduced in the section “Predictor Variables Included in Our Data Set” to build our ML models. Table 3 displays the best RMSE values obtained from each of the models for each of the clinics when we included the engineered predictors. For each clinic (ie, each row), the best performing method is italicized. As different clinics followed different operational schemes and their wait times had different profiles, there was not a single ML model that outperformed all of the rest across all clinics. However, the random forest method appeared to be the most promising method as it provided the minimum RMSE values for 4 (out of the 8 clinics) and the second minimum RMSE for the other 4 clinics. It is notable that the linear regression method, regardless of its simplicity, outperformed other ML methods at some clinics such as Women’s Clinic. This can be attributed to the existence of linear patterns in the data [39], having small sample sizes [40], and the fact that the grid search method provides an optimal combination of the selected subset of hyperparameter values, but it cannot guarantee the global optimality of the output [13].

**Table 3:** Comparison of the root mean square error of different machine learning methods^a^.

Clinic	Linear regression	Random forest	K-nearest neighbors	Support vector machine	Neural network	Decision tree
Anxiety and Mood Disorders Clinic	66.88	*49.89*	70.37	52.64	83.44	50.65
Traumatic Stress Clinic	94.02	93.54	98.95	*86.6*	108.36	102.71
Borderline Personality Self-Regulation Clinic	50	*49.47*	51.94	50.61	61.98	56.16
Women’s Clinic	*33.45*	36.16	42	39.63	46.93	56.73
Prompt Care Clinic	19.04	*16.49*	16.83	17.13	16.87	17.13
Prompt Anxiety and Mood Disorders Consultation	121.25	119.6	125.64	*117.83*	142.53	131.28
Prompt Transitional Aged Youth Consultation	29.82	26.19	26.3	*23.56*	28.92	28.34
Prompt Adolescent Consultation	20.4	*18.04*	31.8	19.13	26.6	20.84

^a^For each clinic (ie, each row), the best performing method is italicized.

### Hyperparameter Tuning

Table 4 displays the list of hyperparameters that we used to tune each of the ML methods, the range of values for each hyperparameter, and the selected values for the AMD clinic. The selected values varied across clinics.

Table 5 displays the selected values of the random forest hyperparameters across different clinics. For some settings, the neural network method with 1 hidden layer could be the same as the linear regression method [12]; to avoid duplications, we did not consider such settings for the neural network method.

**Table 4:** Hyperparameters used for tuning the machine learning methods and their selected values for the Anxiety and Mood Disorders Clinic.

Machine learning method, parameter		Range	Selected value	Explanation
Linear regression		N/A^a^	N/A	N/A
**Random forest**				
	mtry	1 to 20	16	Number of predictors at each split
	min_n	2 to 40	14	Minimum node size
**K-nearest neighbors**				
	neighbors	1 to 15	13	Number of neighbors to consider
	dist_power	0.1 to 2	0.21	Minkowski distance parameter
	weight_func	—^b^	Rectangular	Kernel function for weighting sample distribution
**Support vector machine**				
	cost	2^–10^ to 2^5^	22.31	The cost of wrong predictions
	rbf_sigma	10^–10^ to 10^0^	10^–1.76^	Radial basis function parameter
	margin	0 to 0.2	0.11	Epsilon for support vector machine insensitive loss function
**Neural network**				
	hidden_units	1 to 10	9	Number of units in the hidden model
	penalty	10^–10^ to 10^0^	10^–0.39^	Amount of weight decay
	epochs	10^1^ to 10^3^	993	Number of training iterations
**Decision tree**				
	cost_complexity	10^–10^ to 10^–1^	10^–8.02^	Cost/complexity parameters
	tree_depth	1 to 15	3	Maximum depth of the tree
	min_n	2 to 40	17	Minimum node size

^a^N/A: not applicable.

^b^triweight, triangular, rectangular, rank, optimal, inv, gaussian, epanechnikov, cos, biweight.

**Table 5:** Hyperparameters of the random forest method across different clinics.

Clinic	Count of splitting variables	Count of trees	Minimal node size
Anxiety and Mood Disorders Clinic	16	1000	14
Traumatic Stress Clinic	17	1000	29
Borderline Personality Self-Regulation Clinic	2	1000	31
Women’s Clinic	17	1000	39
Prompt Care Clinic	17	1000	39
Prompt Anxiety and Mood Disorders Consultation	19	1000	30
Prompt Transitional Aged Youth Consultation	11	1000	10
Prompt Adolescent Consultation	11	1000	10

### Variable Importance

**Figure 3:**
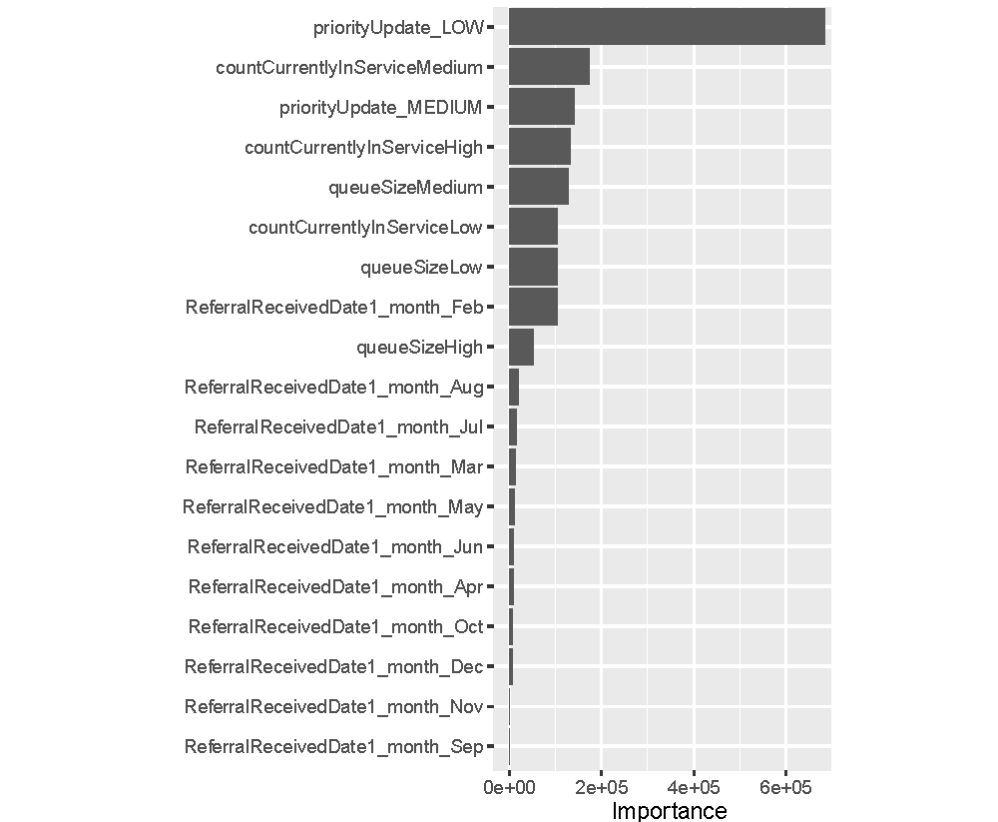
Importance of the predictor variables, measured by impurity (variance of the responses), at the Anxiety and Mood Disorders Clinic.

The random forest method provides measures of importance for predictor variables. These measures of importance help the user to identify variables that have the most and the least impacts on the outcome variable. Figure 3 displays the importance of predictor variables, measured by impurity (variance of the responses) at the AMD Clinic. The rankings of the importance of predictor variables were similar to those shown in Figure 3 at other clinics. According to Figure 3, *priorityUpdate*, *countCurrentlyInService*, and *queueSize* were the most influential variables. In our models, *priorityUpdate* variables denoted the last priority assigned to each patient, *countCurrentlyInService* variables denoted how many patients of different priority levels were currently receiving service (ie, were in the follow-up queues) at the referral time, and *queueSize* variables denoted how many patients of different priority levels were waiting in the primary queue at the referral time. The seasonality variables did not play important roles in wait time prediction.

## Discussion

### Current State

Although operations management and ML tools have been widely used in different sectors of care for physical diseases to improve the waiting list management, there has not been such studies in the mental health care sector. Previous research has demonstrated positive effects of operational and policy improvements that have taken place to improve the wait list management in physical health care, for example, cancer care [41]. In 2015, the Canadian Wait Time Alliance [42] reported that although there had been significant progress in the wait time management in 5 areas of focus in the 2004 Health Accord (hip and knee replacement, cataract, bypass surgery, radiation therapy, and diagnostic imaging), mental health care struggled with long waiting times and required immediate attention nationwide. That report also outlined that universal measures did not even exist to track access to psychiatric care across the country. Loebach and Ayoubzadeh [43] compared the waiting times for psychiatric patients and patients with physical problems in the province of Ontario, Canada, and concluded that while the former group often ended up waiting beyond the target waiting times specified by the province, the latter group often received their treatments within the target time window.

### No-show Rates

**Figure 4:**
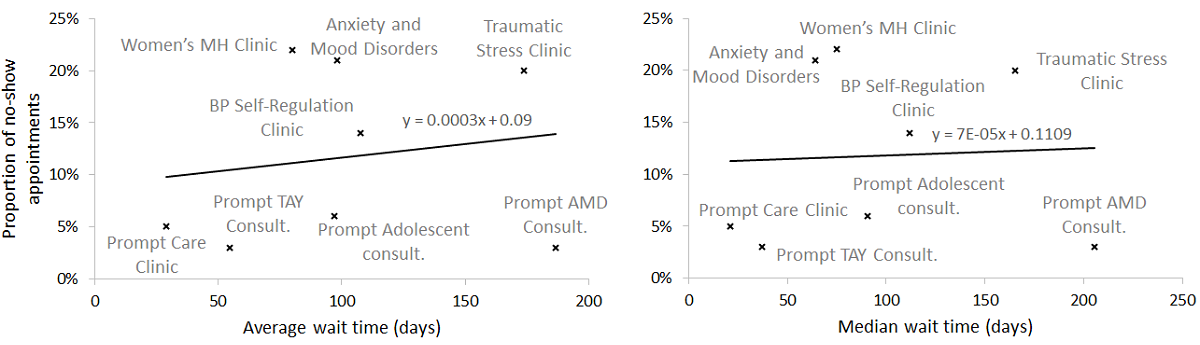
Correlation between no-show appointments and wait times. AMD: Anxiety and Mood Disorders; BP: Borderline Personality; Consult.: Consultation; MH: Mental Health; TAY: Transitional Aged Youth.

In addition to adverse impacts that long wait times have on patients’ health conditions, they also cause higher no-show rates that cause operational complications for health care managers [44,45]. The no-show rate may depend on factors such as wait time and quality of care and may vary between 5% and 80% in different health care sectors [44,45]. Figure 4 illustrates the positive correlation between longer wait times and higher no-show rates in our data, which indicates that shortening wait times may decrease the no-show rates as well.

### Random Forest Method

In this research, we applied 6 different ML methods to predict wait times in mental health care and to identify factors that drive the long wait times. The input data were highly deidentified, which limited the data utility. The random forest method enhanced by the system’s knowledge turned out to be the most promising method. The good performance of this model could be attributed to some appealing computational features of the random forest method, including its low sensitivity to outliers and its ability in capturing complex interactions between predictor variables [46]. From a practical point of view, another appealing feature of the random forest method is its relatively low sensitivity to parameter tuning [46]. Identifying the random forest method as a superior ML method to predict wait times is consistent with findings of [47] that identify this method as the most accurate method to calculate the probability of waiting more than 1 day before receiving treatment for patients with opioid use disorder.

### Managerial Insights

The impurity measure of variable importance, which was the basis for ranking predictive variables in Figure 3, indicates that the total amount of increase in the mean square error of the model output resulted from a random permutation of each variable in the test set. According to Figure 3, the long wait times can be attributed to the usage of the priority system in assigning the care resources to patients. In priority systems, low-priority patients are preceded by patients from higher priority levels and may end up waiting an extended period of time to receive a relatively simple treatment. It is likely that during their long wait times, low-priority patients are changed to higher-priority patients owing to condition deterioration. This phenomenon has been observed in other health care sectors, and using the “fast-track” system for low-priority patients has been suggested as a potential solution [48-50]. In a fast-track system, the waiting line is broken into 2 separate lines: one for low-priority patients and one for patients with higher priorities. One potential advantage of this approach is that because of the simpler nature of care required by low-priority patients, they can be attended by “less-trained” clinicians, freeing up the “more-trained” clinicians for patients with more complex needs. One potential disadvantage of the fast-track system is that the improvement in waiting time of low-priority patients may come at the cost of longer wait times for patients with higher priorities.

### Limitations

Small sample sizes coupled with very large variations within wait times of each clinic imposed the main limitation in this study. There was also a significant difference between the wait time profiles of clinics such that the generalized models for all clinics performed poorly in comparison to models for individual clinics. In addition, the following approximations also impacted the accuracy of model predictions.

1. Care Resource Capacity Limitation: There was no access to the real capacity offered to patients at each clinic at a given day and therefore, we created proxy variables to approximate the capacity.
2. Group Meeting Limitation: Of the clinics that we reviewed within Ontario Shores, the AMD, Traumatic Stress, Borderline Personality Self-Regulation, and Women’s clinics provided care through group meetings where multiple patients attend at the same time. The dynamics of group visits in clinics that provide group treatments were not clear and therefore, we could not explicitly capture the potential impacts of these treatments on wait times.

### Conclusion

In this study, we used retrospective highly deidentified administrative data from 8 clinics at Ontario Shores to build 6 different ML models to predict wait times. We enhanced our models by system knowledge to mitigate the limiting impact of deidentification on our data utility. The data included 4187 patients who received care through 30,342 appointments. The random forest method provided the minimum RMSE values for 4 of the 8 clinics and the second minimum RMSE for the other 4 clinics. The priority system was identified as a factor that contributed to long wait times, and a fast-track system was suggested as a potential solution. Despite the challenges with the wait time source data, this research provided Ontario Shores with a deeper understanding of the extent of and contributors to their wait times on a clinic-by-clinic basis. This research provided Ontario Shores with information and knowledge to pursue quality improvement initiatives to reduce wait times.

## Abbreviations

AMD: Anxiety and Mood Disorders
ML: machine learning
RMSE: root mean square error
